# Validation of the Subjective Happiness Scale, Oxford Happiness Questionnaire and Pemberton Happiness Index among women with breast cancer in a multi-ethnic Asian setting

**DOI:** 10.3332/ecancer.2025.1873

**Published:** 2025-03-13

**Authors:** Nithiya Sinarajoo, Yek-Ching Kong, Ranjit Kaur, Ros Suzanna Bustamam, Nur Fadhlina Abdul Satar, Sharminii Jaya-Prakason, Harenthri Devy Alagir Rajah, Mahmoud Danaee, Cheng-Har Yip, Nirmala Bhoo-Pathy

**Affiliations:** 1 Department of Social and Preventive Medicine, Faculty of Medicine, Universiti Malaya, Kuala Lumpur 50603, Malaysia; 2 Breast Cancer Welfare Association (BCWA), 16 Jalan Utara, Petaling Jaya 46200, Selangor, Malaysia; 3 Department of Radiotherapy and Oncology, Hospital Kuala Lumpur, Kuala Lumpur 50586, Malaysia; 4 Department of Oncology, University Malaya Medical Centre, Kuala Lumpur 59100, Malaysia; 5 Subang Jaya Medical Centre, Subang 47500, Selangor, Malaysia; a https://orcid.org/0000-0003-0568-8863

**Keywords:** breast cancer, happiness, questionnaire validation, Subjective Happiness Scale, Oxford Happiness Questionnaire, Pemberton Happiness Index

## Abstract

**Background:**

It is uncertain if the current tools that are used to measure happiness in the general population are valid in women with breast cancer.

**Objective:**

We determined the psychometric properties of the Subjective Happiness Scale (SHS), Oxford Happiness Questionnaire (OHQ) and Pemberton Happiness Index (PHI) among women with breast cancer in a multi-ethnic Asian setting.

**Methods:**

The internal consistency and construct validity of the SHS, OHQ and PHI were assessed. Criterion validity was determined by measuring the correlations of the study tools with the relevant domains of the European Organisation for the Research and Treatment of Cancer Quality of Life Questionnaire 30 and the Depression, Anxiety and Stress Scale.

**Results:**

Cronbach’s alpha of the SHS, OHQ and PHI ranged from 0.75 to 0.92. In the exploratory factor analyses, a one-factor model was recommended for SHS and PHI. A two-factor model was recommended for OHQ. Confirmatory factor analysis indicated that the two-factor model for OHQ demonstrated acceptable convergent and divergent validity with composite reliability >0.70. Both OHQ and SHS revealed a moderately positive correlation with health-related quality of life, and a moderately negative correlation with psychological distress.

**Conclusion:**

This study demonstrated that SHS and OHQ are valid and reliable tools to measure happiness among women with breast cancer in multi-ethnic Asian settings. While the PHI seems promising, we were unable to confirm its criterion validity in the current study.

## Introduction

Higher levels of education, rising consumerism and increased access to information over the past decades have altered the expectations of healthcare delivery [1]. Health systems around the world are now expected to adopt more humanistic approaches in delivering health care, where patients are viewed holistically and respected as whole persons. Within the context of cancer care, a myriad of patient-centered outcomes corresponding to various domains of health including physical, mental, psychosocial and financial well-being, have been proposed and investigated [2]. Notably, mental well-being has been recognised as an important domain in cancer care given its influence on patients’ attitudes and subsequent coping mechanisms toward their illness, cancer management and living with the disease [3]. While mental health has traditionally been gauged by measuring the absence of mental disorders such as anxiety and depression, increasing theoretical and empirical evidence in healthy individuals suggests that positive psychology, which focuses on positive psychological states, positive psychological traits, positive relationships and positive institutions are also important as they have been associated with better health and longevity [4].

Happiness, which is often referred as subjective well-being in the scientific literature may serve as an important construct in positive psychology [5]. It is defined as the evaluation of a person’s life quality from that individual’s own perspective, where it is thought that different individuals are likely to weigh objective circumstances in their lives differently depending on their own goals, values and culture [6]. Components of subjective well-being are affect, hedonic well-being and eudemonic well-being [7].

The pursuit of happiness has been stipulated as an important human goal by the United Nations since 2011 and has been touted as an indicator to evaluate the effectiveness of public policies by governments worldwide [8]. However, happiness or subjective well-being is not commonly evaluated in clinical practice or research, with many clinicians and scholars relying on quality-of-life measurements to gauge the well-being of patients [9]. While assessment of health-related quality of life (HRQoL) following cancer is useful for evaluating symptoms and functional well-being, it may miss non-health-related aspects of life that are equally important to patients [10]. 

Prior research in women with early breast cancer for instance shows that patients who reported being happy were associated with higher physical functioning and lower symptom burden, possibly due to enhanced emotional resilience and coping skills [11]. Also, women with recurrent breast cancer who reported experiencing more joy were significantly associated with higher survival rates [12]. It has therefore been posited that happiness may exert its positive effects on overall well-being and survival following cancer via both biological and behavioural pathways. A previous study for instance has demonstrated that a happier state of mind in women was associated with reduced levels of inflammatory markers [13]. Likewise, happiness may positively influence psychological adjustment [14], lifestyle behaviours and stress management, collectively contributing to improved overall well-being in women with breast cancer [11]. It is also conceivable that in some individuals, a traumatic life event such as being diagnosed with breast cancer may foster post-traumatic growth that aids patients in navigating the ensuing emotional challenges and thriving with cancer [15], potentially leading to higher levels of happiness along their cancer journey. Nonetheless, more research is needed to assess the construct of happiness in the context of cancer and to evaluate its role as a predictor of health outcomes, or as a patient-centred outcome in routine oncology practice. A first step however is to identify valid tools to measure happiness in individuals with cancer.

Previous research validating happiness questionnaires to this end has primarily relied on the general population, which limits their usefulness for understanding happiness in clinical populations like women with breast cancer. Central to this discussion is that women with breast cancer might redefine happiness due to their experiences, i.e., by finding joy in smaller things, focusing on resilience and acceptance or prioritising different aspects of life compared to the general population. This shift in perspective may affect how they respond to the existing questionnaires that measure happiness. We therefore sought to validate the Subjective Happiness Scale (SHS) [16], Oxford Happiness Questionnaire (OHQ) [17] and Pemberton Happiness Index (PHI) [18] among women living with and beyond breast cancer in a multi-ethnic, multicultural Asian setting.

## Methods

Given the conceptual overlap between the dimensions of happiness and subjective well-being, the terms are often used interchangeably in the literature [19, 20]. As this notion is corroborated by empirical evidence [21], we have used the terms happiness and subjective well-being interchangeably in the present work.

In the context of cancer, several instruments have been commonly used to measure subjective well-being, such as the SHS [16], OHQ [17], PHI [18] and Satisfaction with Life Scale (SWLS) [22] (Supplementary Table). Based on the current study’s conceptual framework, which was adapted from Diener and Ryan’s Tripartite Model of Subjective Well-being as well as Galinha and Pais-Ribeiro’s proposal of using cognitive (e.g., patient’s personal beliefs/values, health beliefs), affective (patients’ positive or negative traits) and contextual predictors (socio-demographic characteristics, life circumstances) to assess subjective well-being [23, 24], we have selected the SHS, OHQ, PHI for further validation, leaving out SWLS, which is focussed on life satisfaction but does not measure other relevant constructs [22].

### Study tools

#### Subjective Happiness Scale

The SHS is a broadly stated 4-item self-reported measure that assesses an individual’s overall subjective well-being [16]. The scale demonstrates a unitary structure with high reliability (Cronbach’s alpha = 0.860) [16] and has been translated into Turkish, Spanish, Lebanon and Greek languages and validated in the general population in the respective settings [25–27]. Furthermore, it has also been used to measure happiness in clinical studies, including in a cross-sectional study of women living with early breast cancer [11]. The response format for SHS is a seven-point Likert-type scale (1: less happy to 7: happier). A single composite score is computed by averaging the responses to the four items following the reverse coding of the fourth item, with higher scores reflecting higher levels of happiness [28].

#### Oxford Happiness Questionnaire

The OHQ consists of 29 similarly worded single items indicating all predictors of well-being, with a standardised six-point Likert-type scale [17]. The scale contains seven subscales namely satisfaction with life, efficacy, sociability/empathy, positive outlook, well-being, cheerfulness and self-esteem. Cronbach’s alpha of the English scale was 0.910 [17]. The OHQ has been translated into Hindi, Chinese and Turkish languages [29–31]. Like SHS, it has also been used in prior studies to measure happiness in patients, including in those with cancer [25–31]. The sum of scores for all the 29 items in OHQ (11 items with reversed scoring) must be divided by 29 to derive the final score that can range between 1 and 6. The score is interpreted as following: 1–2: not happy, 2–3: somewhat happy, 3–4: neutral, 4: somewhat happy, 4–5: rather happy, 5–6: very happy and 6: too happy [17].

#### Pemberton Happiness Index

The PHI has a single structure which measures subjective well-being at different timeframes, namely remembered well-being and experienced well-being [18]. It has been validated in Spain, Germany, Russia, Turkey, India and Japan, with the Cronbach’s alpha ranging between 0.890 and 0.910 [18]. The tool has also been used to measure happiness among cancer patients and caregivers in a study conducted in Brazil [27]. The remembered well-being domain in PHI includes 11 items with a 10-point Likert scale response (0: totally disagree to 10: totally agree), whereas the experienced well-being (positive and negative events that occurred the day before) domain comprises ten items requiring dichotomous response options (yes/no) [18]. The index for experienced well-being is the sum of positive experiences (each counted as ‘1’) and absence of negative experiences (each counted as ‘1’) of the day before, leading to a single overall score ranging between 0 and 10. The sum of the corresponding scores from remembered well-being and experienced well-being must be divided by 12 to produce the total mean score (ranging from 0 to 10). Here, a higher index denotes higher levels of happiness [18].

### Study setting

Malaysia comprises a multilingual and multi-ethnic society. Malaysian culture, a blend of Malay, Chinese, Indian and indigenous influences, holds specific values that likely shape how individuals perceive and experience happiness. Therefore, to ensure the accuracy and cultural relevance, we validated the English versions of SHS, OHQ and PHI.

### Content validity

Content validity of SHS, OHQ and PHI was performed by a panel of six experts from the psychological and public health fields, including two males and four females from the Malay, Chinese and Indian ethnic groups. For each item, the expert panels were prompted with: ‘Is each item relevant to happiness/ subjective well-being?’ whereby the experts were asked to provide ratings of 1 to 4 for each question, with ratings of 3 or 4 indicating agreement. The item content validity index (I-CVI) and the scale content validity index (S-CVI) were determined based on feedback from the experts. The acceptable values for I-CVI and S-CVI were at least 0.790 and 0.800, respectively [32]

### Study population and data collection

A convenience sample of 300 Malaysian women aged 18 and older, diagnosed with stages I–IV breast cancer (any subtype) and proficient in English, was recruited from both rural and urban areas for the field test. To validate the happiness questionnaires, these participants were randomly divided into two groups of 150, with one group assigned to exploratory factor analysis (EFA) and the other to Confirmatory Factor Analysis (CFA). This sample size aligns with the recommended minimum of 150 participants for both EFA and CFA [33, 34].

The study employed a two-pronged recruitment strategy. In the hospital-based approach, eligible participants were identified and recruited face-to-face by trained research staff from the oncology clinic of a major public hospital in Klang Valley, an urban agglomeration in the highly-developed Central Region of Malaysia. In the community-based approach, members of a local breast cancer non-governmental organisation who were residing in various parts of Malaysia were recruited by trained volunteers from within the organisation due to the COVID-19 pandemic. Here, all interviews were telephone-administered. In both approaches, potential study participants were briefed using a participant information sheet. Informed consent was obtained prior to administration of the study questionnaires.

Apart from SHS, OHQ and PHI, participants also answered the European Organisation for Research and Treatment for Cancer Quality of Life Core 30 (EORTC QLQ C30), as well as the Depression, Anxiety and Stress Scale (DASS-21) questionnaires. Socio-demographic data (age, ethnicity, marital status, highest attained education, religion, place of residence and total monthly household income) were also collected.

### Statistical analysis

#### Construct validity – dimensionality analysis

EFA were conducted using the Statistical Package for the Social Sciences (SPSS) version 25, IBM Company, Chicago, IL, USA. The Kaiser-Meyer-Olkin (KMO) test (using a cutoff >0.60) was conducted to ascertain sampling adequacy [35, 36]. Bartlett’s test of sphericity (*p*-value <0.05) [35] was employed to assess the suitability of the correlation matrix for factor analysis. The number of factors extracted was determined by examining the eigenvalues (>1) and plotting the scree plot. Varimax rotation was used as varimax rotation provides a clear and more interpretable structure. Parallel analysis was then performed to determine the optimum number of factors to be retained in the factor analysis using the StatsToDo. The percentage of explained variance was also assessed, and a value of more than 50.0% was considered acceptable [36]. Items with a factor loading of <0.50 were removed [37].

#### Reliability

Cronbach’s alpha and corrected item-total correlation (CITC) were computed using SPSS. Values of at least 0.30 for the CITC and 0.70 for Cronbach’s alpha were deemed acceptable [38].

#### Confirmatory factor analysis

CFA was conducted via the SmartPLS software 4.0 using the partial least squares structural equation modelling. The measurement model was assessed using factor loadings (≥ 0.50) [37], Cronbach’s alpha and composite reliability (CR) (> 0.70) [37, 39]. To assess convergent validity, the average variance extracted (AVE) was calculated. An AVE value greater than 0.4 was considered acceptable, provided that the domain’s CR exceeded 0.60 [40]. Discriminant validity was determined by assessing the Heterotrait-Monotrait ratio (HTMT) <0.90 [41] and cross-loading of indicators (<0.10) [42].

#### Criterion validity – predictive validity

Criterion validity was determined by measuring the correlations of the study tools with the relevant domains from EORTC QLQ C30 and DASS-21 questionnaires using Pearson’s correlation test [43]. 

## Results

### Content validity

The I-CVI and S-CVI values for all the questionnaires were adequate, ranging from 0.83 to 1.00 and 0.92 to 0.98, respectively. (SHS, I-CVI = 0.83–1.00 ,S-CVI = 0.96; OHQ, I-CVI = 0.83–1.00 ,S-CVI = 0.92; PHI, I-CVI = 0.83–1.00,S-CVI = 0.98).

### Field testing of SHS, OHQ and PHI

#### Participant characteristics

Most of the study participants were aged between 41 and 60 years (61.3% *n* = 184). The majority who answered were Malay (43.7%). About 50% of the study participants comprised patients whose highest-attained education was up to secondary school (52.3%). Most participants were from urban (93.0%) areas (Table 1).

#### Construct validity – dimensionality analysis

The KMO values for the items in the SHS, OHQ and PHI were 0.74, 0.90 and 0.87, respectively. The *p*-values for Bartlett’s spherical test were also significant (*p* < 0.001), all suggesting sampling adequacy.

### Subjective Happiness Scale

Only one factor was extracted for SHS with an eigenvalue exceeding one. Parallel analysis further confirmed one factor to be extracted. The total variances explained were acceptable at 70.8% (Table 2).

### Oxford Happiness Questionnaire

Examination of the scree plot indicated two factors to be extracted for OHQ. Parallel analysis further confirmed that a two-factor model is recommended for OHQ. The total variances explained was 50.0%. The factor loadings of items 2,5,10,14,20,27,28,29 was less than 0.50. Hence, the items were dropped from further analysis. The final stage of the EFA required interpretation of the extracted factors. Factor 1 was labelled ‘cheerfulness’, while Factor 2 was labelled ‘optimism’. A total of 15 items were loaded in Factor 1 with Cronbach’s alpha of 0.92 and 6 items in Factor 2 with Cronbach’s alpha of 0.79. The Cronbach’s alpha values were deemed adequate (Table 3).

### Pemberton Happiness Index

Results from the parallel analysis indicated that a one-factor model is recommended for PHI. The factor loadings of items (Section A -10; Section B- 1,2,3,5,6,9) were less than 0.50. Hence, the items were dropped from further analysis. The total variances explained was 51.3% (Table 4)

## Reliability

Internal consistency analysis revealed good reliability of SHS, OHQ and PHI, with Cronbach’s alpha values ranging from 0.83 to 0.92 (Table 5). The CITC ranged from 0.42 to 0.91 for all three questionnaires (Table 5).

## Confirmatory Factor Analysis

### Subjective Happiness Scale

Four items with one factor were included for CFA. The AVE of the model was 0.71. The tool also demonstrated good internal consistency with a CR value of 0.87 and Cronbach’s alpha of 0.86, demonstrating good convergent validity.

### Oxford Happiness Questionnaire

Twenty-one items with two factors were included for CFA. The factor loadings of items 3 and 18 were lower than 0.50; thus, the items were dropped and the model was rerun. (Table 6) The AVE of the rerun model was 0.49. The tool also demonstrated good internal consistency with a CR value of 0.92 and Cronbach’s alpha 0.91, demonstrating good convergent validity. The HTMT ratios were 0.56 for the outer model domains, with no cross-loadings between the items, demonstrating good discriminant validity.

### Pemberton Happiness Index

The one factor was included for CFA. The AVE of the model was 0.47. The tool also demonstrated good internal consistency with a CR value of 0.87 and Cronbach’s alpha of 0.88, demonstrating good convergent validity.

## Criterion validity

### Subjective Happiness Scale

The SHS displayed significant and moderate positive correlations with HRQoL (*r*: 0.57). In line with this, significant and moderate negative correlations were observed with psychological distress (*r*: –0.56).

### Oxford Happiness Questionnaire

Additionally, OHQ exhibited significantly moderate positive correlations with HRQoL (*r*: 0.48). Likewise, for psychological distress, significant and moderate negative correlations were noted (*r*: –0.64).

### Pemberton Happiness Index

However, PHI was weakly correlated with HRQoL (*r*: 0.14) and with psychological distress (*r*: – 0.07). The results were not statistically significant.

### Correlations among SHS, PHI and OHQ

The OHQ and SHS were significantly and moderately correlated (*r* = 0.56). However, neither SHS (*r* = 0.11) nor OHQ (*r* = 0.16) demonstrated significant correlations with PHI.

## Discussion

Our validation exercise revealed that both the English version of the SHS and OHQ are valid and reliable tools for measuring happiness/subjective well-being among women living with and beyond breast cancer in the Malaysian setting. The notion that happy cancer survivors are more likely to report better general health status and quality of life [11] is corroborated by the current study results, in which both OHQ and SHS were positively correlated with HRQoL but negatively linked with psychological distress. Although the PHI was found to possess adequate construct validity and reliability, we failed to demonstrate its criterion validity in the present study. It is thought that this may be due to differences in the timeframe covered by the EORTC QLQ C30 and DASS-21 questionnaires, both of which measured the patients’ experiences in the past week, compared to the PHI that accounted for patients’ happiness in both the immediate term (the day of questionnaire administration) as well as in the long-term.

The one-factor model extracted for SHS and PHI in the present study is similar to that found in previous validation studies conducted among the general population in other settings [18, 44, 45]. The number of extracted factors for OHQ in this present study also differed from those of other translated versions, including the Hindi and Turkish versions [30, 31]. The above may be attributed to cultural differences between these populations, who may hold different beliefs and practice different religions, all of which could influence their perception of happiness [46]. Apart from the above, prior evidence has also highlighted the influence of a country’s level of development in shaping the subjective well-being of its people, which may also explain the disparities in the findings of studies conducted across different countries [47]. These findings highlight the complexity of conducting cross-cultural research. It is imperative that researchers remain cognizant of the pervasive influence of culture, including religion, as well as resource settings on psychological constructs.

### Strengths and limitations

Our study also included women living with breast cancer from different walks of life, whereby women of varying the age, ethnicity, religious background and income status were recruited. It is nonetheless acknowledged that the number of participants from the rural areas was limited. While the PHI seems promising, we were unable to confirm its criterion validity in the current study. Future studies may need to identify instruments that measure HRQoL, psychological distress or other related constructs within the same timeframe as the PHI. Furthermore, future studies may examine the interaction between stages of breast cancer and questionnaire responses.

### Implications on practice

Besides potentially acting as a prognostic predictor of breast cancer outcomes due to its influence on various physiological, psychological and behavioral factors, happiness in itself may function as a patient-centered outcome. Measuring happiness in women with breast cancer may facilitate in uncovering insights into post-traumatic growth, understanding its impact on happiness and developing effective interventions to support patients’ psychological adjustment and overall well-being throughout their breast cancer journey. Assessment of happiness within clinical settings and community settings may also guide optimisation of supportive and survivorship services that meets the needs of patients and their caregivers. Likewise, happiness or subjective well-being could also serve as a patient-centred endpoint in clinical trials [48]. From the health systems perspective, subjective well-being may complement other measures of well-being including social well-being and financial well-being, to guide policymakers and health systems administrators in evaluating public policies and health policies, and be used in performing cost-benefit analyses.

Based on the current study findings, it is suggested that the OHQ is used in studies where happiness or subjective well-being is the main exposure (or predictor) or the main outcome of interest. On the other hand, the SHS, which is a relatively shorter and simpler tool may be useful in studies where happiness is one of the covariates that is being studied.

## Conclusion

The English version of the OHQ and SHS are valid and reliable tools to measure happiness (or subjective well-being) among women diagnosed with breast cancer in multi-ethnic Asian settings. Happiness measures provide valuable insights into patients’ subjective experience of their illness and treatment. Incorporation of routine screening of happiness in oncology practices has the potential to enable healthcare teams to deliver care that is responsive to the needs of patients, i.e., patient-centred care. These tools can also be used by various stakeholders including policymakers and civil societies in evaluating value in cancer care from the patients’ perspectives.

## Conflicts of interest

The authors declare no conflicts of interest.

## Funding

This study was funded by the Ministry of Higher Education Malaysia (IIRG006C-19HWB).

## Ethics approval

This study received ethics approval from the Malaysian Medical Research and Ethics Committee (MREC) (NMRR-20-3192-56840).

## Author contributions

The authors confirm contribution to the paper as follows: Study conception and design: Nirmala Bhoo-Pathy; Data collection: Nithiya Sinarajoo, Yek-Ching Kong, Ranjit Kaur, Ros Suzanna Bustamam, Nur Fadhlina Abdul Satar & Sharminii Jaya-Prakason; Analysis and interpretation of results: Mahmoud Danaee, Nithiya Sinarajoo, Yek-Ching Kong & Nirmala Bhoo-Pathy; Draft manuscript preparation: Nithiya Sinarajoo, Yek-Ching Kong & Nirmala Bhoo-Pathy. All authors reviewed the results and approved the final version of the manuscript.

## Figures and Tables

**Table 1.** Characteristics of study participants.

Characteristic	Overall *N* = 300 *n* (%)	Exploratory factor analysis *n* = 150 *n* (%)	Confirmatory factor analysis *n* = 150 *n* (%)
Age (years)			
<40	41 (13.7)	19 (12/7)	22 (14.7)
41–60	184 (61.3)	97 (64.7)	87 (58.0)
>61	75 (25.0)	34 (22.7)	41 (27.3)
Region			
Urban	279 (93.0)	140 (93.3)	139 (92.7)
Rural	21 (7.0)	10 (6.7)	11 (7.3)
Ethnicity			
Malay	131 (43.7)	39 (26.0)	92 (61.3)
Chinese	126 (42.0)	88 (58.7)	38 (25.3)
Indian	31 (10.3)	15 (10.0)	16 (10.7)
Others	12 (4.0)	8 (5.3)	4 (2.7)
Religion			
Islam	134 (44.7)	42 (28.0)	92 (61.3)
Buddhism	88 (29.3)	52 (34.7)	36 (24.0)
Hinduism	25 (8.3)	11 (7.3)	14 (9.3)
Christianity	39 (13.0)	35 (23.3)	4 (2.7)
Others	14 (4.7)	10 (6.7)	4 (2.7)
Marital status			
Married	229 (76.3)	122 (81.0)	107 (71.3)
Single	39 (13.0)	21 (14.0)	18 (12.0)
Widowed	22 (7.3)	6 (4.0)	16 (10.7)
Divorced	9 (3.0)	1 (0.7)	8 (5.3)
Others	1 (0.3)	0 (0.0)	1 (0.7)
Monthly household income ^†^			
Low	150 (50.0)	44 (29.3)	106 (70.7)
Middle	101 (33.7)	72 (48.0)	29 (19.3)
High	49 (16.3)	34 (22.7)	15 (10.0)
Highest attained education level			
Secondary or lower	157 (52.3)	72 (48.0)	85 (56.7)
Diploma or pre-university	60 (20.0)	31 (20.7)	29 (19.3)
Tertiary	83 (27.7)	47 (31.4)	36 (24.0)

† Based on classification of the Department of Statistics Malaysia 2019

**Table 2.** Items loaded for SHS.

Items	Factor loading	Eigenvalue	Variance (%)	Cumulative variance (%)
**English**		**2.83**	**70.77**	**70.77**
1) In general, I consider myself:	0.89			
2) Compared to most of my peers, I consider myself:	0.90			
3) Some people are generally very happy. They enjoy life regardless of what is going on, getting the most out of everything. To what extent does this characterization describe you?	0.87			
4) Some people are generally not very happy. Although they are not depressed, they never seem as happy as they might be. To what extent does this characterization describe you?	0.69			

**Table 3.** Items loaded for OHQ.

Items	Factor loading	Eigenvalue	Variance (%)	Cumulative variance (%)
Factor 1 (cheerfulness)		8.294	40.0	40.0
3 (I feel that life is very rewarding)	0.69			
4 (I have very warm feelings towards almost everyone)	0.52			
7 (I find most things amusing)	0.50			
8 (I am always committed and involved)	0.63			
9 (Life is good)	0.59			
11 (I laugh a lot0)	0.75			
12 (I am well satisfied about everything in my life)	0.58			
15 (I am very happy)	0.77			
16 (I find beauty in some things)	0.67			
17 (I always have a cheerful effect on others)	0.78			
18 (I can fit in (find time for) everything I want to)	0.62			
21 (I feel fully mentally alert)	0.74			
22 (I often experience joy and elation)	0.68			
25 (I feel I have a great deal of energy)	0.56			
26 (I usually have a good influence on events)	0.70			
Factor 2 (Optimistim)		2.23	10.6	50.0
1( I don't feel particularly pleased with the way I am)	0.63			
6 (I am not particularly optimistic about the future)	0.74			
13 (I don't think I look attractive)	0.57			
19 (I feel that I am not especially in control of my life)	0.76			
23 (I don't find it easy to make decisions)	0.65			
24 (I don't have a particular sense of meaning and purpose in my life)	0.68			

**Table 4.** Items loaded for PHI.

Items	Factor loading	Eigenvalue	Variance (%)	Cumulative variance (%)
**English**		**5.12**	**51.25**	**51.25**
1) I am very satisfied with my life	0.68			
2) I have the energy to accomplish my daily tasks.	0.67			
3) I think my life is useful and worthwhile.	0.75			
4) I am satisfied with myself.	0.74			
5) My life is full of learning experiences and challenges that make me grow.	0.71			
6) I feel very connected to the people around me.	0.65			
7) I feel able to solve the majority of my daily problems.	0.78			
8) I think that I can be myself on the important things.	0.77			
9) I enjoy a lot of little things every day.	0.75			
11) I think that I live in a society that lets me fully realize my potential.	0.64			
12) Total score of Section B^†^	0.70			
F) I was worried about personal matters				
G) I learned something interesting				
H) I gave myself a treat				
J) I felt disrespected by someone				

† Ten items in section B are summed (each counted as ‘1’) leading to a single overall score ranging between 0 and 10

**Table 5.** Internal reliability of the SHS, OHQ and PHI in women with breast cancer in Malaysia.

Questionnaires Indices	SHS	OHQ	PHI
Range of CITC	0.53–0.75	0.41–0.82	0.46–0.91
Range of Cronbach’s alpha if the item deleted	0.75–0.89	0.89–0.91	0.91–0.93
Actual Cronbach’s alpha	0.83	0.90	0.92

**Table 6.** Convergent validity of OHQ.

Domain	Item	Initial model	Modified model	Cronbach’s alpha	CR (rho_a)	AVE
Cheerfulness	3	0.48	Deleted	0.91	0.92	0.49
	4	0.56	0.53			
	7	0.74	0.73			
	8	0.54	0.56			
	9	0.71	0.72			
	11	0.73	0.73			
	12	0.71	0.72			
	15	0.80	0.82			
	16	0.61	0.61			
	17	0.73	0.73			
	18	0.46	Deleted			
	21	0.68	0.68			
	22	0.81	0.81			
	25	0.64	0.67			
	26	0.73	0/73			
Optimism	1	0.53	0.52	0.74	0.78	0.43
	6	0.54	0.54			
	13	0.77	0.76			
	19	0.75	0.75			
	23	0.58	0.59			
	24	0.74	0.74			

## Supplement

**Supplementary Table.** Summary of prior studies measuring happiness in individuals with cancer using various tools.

No.	Year	Author(s)	Paper title	Tool
1	2010	Visser *et al* [S1]	Spirituality and well-being in cancer patients: A review	SHS
2	2017	Kang *et al* [S2]	Who are happy survivors? Physical, psychosocial, and spiritual factors associated with happiness of breast cancer survivors during the transition from cancer patient to survivor	SHS
3	2016	Dowlatabadi *et al* [S3]	The effectiveness of group positive psychotherapy on depression and happiness in breast cancer patients: A randomised controlled trial	OHQ
4	2017	Mirzazadeh and Pirkhaefi [S4]	The effectiveness of clinical creativity therapy model in improving hope and happiness of the patient with breast cancer	OHQ
5	2020	Kondori Fard *et al* [S5]	The effect of hope therapy-based training on the happiness of women with breast cancer: A quasi-experimental study	OHQ
6	2017	Abolghsemi [S6]	The efficacy of emotional intelligence teaching and coping strategies for stress training on general health and happiness of cancer patients	OHQ
7	2018	Ahmadidarrehsima *et al* [S7]	An evaluation of happiness and factors affecting it in patients diagnosed with breast cancer	OHQ
8	2020	Riklikienė *et al* [S8]	Spiritual well-being of cancer patients: What health-related factors matter?	OHQ
9	2022	Kalroozi *et al* [S9]	Comparing the effect of emotional freedom technique on sleep quality and happiness of women undergoing breast cancer surgery in military and nonmilitary families: A quasi-experimental multicenter study.	OHQ
10	2020	de Camargos *et al* [S10]	An explorative analysis of the differences in levels of happiness between cancer patients, informal caregivers, and the general population	PHI
11	2023	Chen *et al* [S11]	Measuring the Wellbeing of Cancer Patients with Generic and Disease-Specific Instruments.	SWLS

Abbrevation : SHS = Subjective Happiness Scale, OHQ=Oxford Happiness Questionnaire, PHI= Pemberton Happiness Index
